# Role of Radiomics to Predict Malignant Transformation of Sinonasal Inverted Papilloma: A Systematic Review

**DOI:** 10.3390/cancers17132175

**Published:** 2025-06-27

**Authors:** Caitlin Waters, Avinash Deshwal, Tom O. Cuddihy, Holly Jones, Hugo C. Temperley, Hannah Kaye-Coyle, Niall J. O’Sullivan, Benjamin M. Mac Curtain, Michael E. Kelly, Orla Young

**Affiliations:** 1 Department of Otolaryngology-Head and Neck Surgery, Galway University Hospital, H91 YR71 Galway, Ireland; 2 Department of Surgery, Fiona Stanley Hospital, Perth 6150, Australia; 3 Department of Radiology, St. James’s Hospital, D08 NHY1 Dublin, Irelandnosulli7@tcd.ie (N.J.O.); 4 Trinity St. James’s Cancer Institute, St. James’s Hospital, D08 NHY1 Dublin, Ireland; kellym11@tcd.ie; 5 Royal College of Surgeons, D02 YN77 Dublin, Ireland; benjaminmaccurtain@rcsi.ie; 6 Department of Surgery, St. James’s Hospital, D08 NHY1 Dublin, Ireland

**Keywords:** radiomics, radiogenomics, oncology, inverted papilloma, sinonasal

## Abstract

Introduction: Sinonasal inverted papilloma is a benign but aggressive tumour of the sinonasal tract. It has the potential for malignant transformation into sinonasal squamous cell carcinoma. Radiomics, which involves the extraction and analysis of quantitative imaging features, has emerged as a promising tool in prediction of tumour behaviour. This systematic review aims to critically evaluate the current literature on the application of radiomics in predicting the malignant transformation of sinonasal inverted papilloma. Methods: A comprehensive literature search was conducted across Medline (via PubMed), EMBASE, and Web of Science. Studies investigating the use of radiomics to predict malignant transformation in sinonasal inverted papilloma were selected based on predefined inclusion criteria. Methodological quality and risk of bias were assessed using the QUADAS-2 tool and the Radiomics Quality Score (RQS). Results: Five studies were included, encompassing 837 participants. All studies were retrospective and utilised MRI-based radiomics in the construction of their models. The radiomic models demonstrated satisfactory predictive performance. The median AUCs across the included studies were 0.954 (range: 0.901–0.987) in the training set and 0.914 (range: 0.8–0.989) in the validation set. Conclusions: This systematic review highlights the potential of radiomics as a predictive tool for the malignant transformation of sinonasal inverted papilloma. Radiomics shows promise as a non-invasive adjunct for clinical decision-making. However, further research is needed to standardise methodologies and validate these findings in larger multicentre cohorts.

## Simple Summary

Inverted papillomas are a type of tumour that grows in the nose and nearby sinuses. Although these tumours are usually not cancerous, they can behave aggressively and sometimes change into cancer. It is difficult for doctors to know in advance which of these tumours might turn into cancer. This study looked at a new method called radiomics, which uses computer programs to analyse medical images, such as MRI scans, in great detail. These programs can find patterns in the images that may not be visible to the human eye. We reviewed five research studies to see if radiomics could help doctors predict which tumours are more likely to become cancer. The results showed that radiomics models were able to do this with a high level of accuracy, especially when used together with clinical information. This means that radiomics could become a helpful, non-invasive tool for planning treatment and follow-up care. However, more research is needed to test these models in larger and more varied patient groups. If confirmed, this approach could help personalise care, avoid unnecessary treatments, and improve outcomes for patients with these tumours.

## 1. Introduction

Sinonasal inverted papilloma (IP) is a rare epithelial neoplasm arising within the nasal cavity and paranasal sinuses. It exhibits histopathologically benign characteristics, yet demonstrates clinically aggressive behaviour marked by local invasiveness, high recurrence rates, and a well-documented propensity for malignant degeneration into sinonasal squamous cell carcinoma (SNSCC) [1]. Despite constituting a minority of sinonasal tumours, sinonasal IP’s clinical relevance is amplified by its unpredictable oncogenic potential, with 5–10% of cases undergoing malignant transformation [2,3,4]. The timely stratification of sinonasal IP lesions at elevated risk of synchronous malignancy or metachronous carcinomatous evolution following resection is critical to inform risk-adapted therapeutic paradigms, including surgical margin optimization, adjuvant intervention, and surveillance protocols.

Advancements in computational imaging analytics have positioned radiomics as a transformative modality in oncological diagnostics, leveraging high-throughput extraction of subvisual, high-dimensional data from conventional imaging to quantify tumour phenotypes. By interrogating features such as textural heterogeneity, morphometric complexity, intensity distribution, and spatiotemporal heterogeneity, radiomic frameworks enable the derivation of latent biomarkers reflective of underlying tumour biology and genomic dynamics [5,6]. Applied to sinonasal IP, radiomics may offer non-invasive risk stratification by identifying imaging signatures predictive of malignant progression, thereby refining preoperative decision-making and personalised therapeutic algorithms. This systematic review and meta-analysis seeks to consolidate and critically evaluate existing evidence on the diagnostic and prognostic utility of radiomic models in anticipating malignant transformation of sinonasal IP, with implications for advancing precision medicine in sinonasal oncology.

## 2. Methods

### 2.1. Study Design and Reporting Guidelines

This study is a systematic review of non-randomised trials and follows the Preferred Reporting Items for Systematic Reviews and Meta-Analyses (PRISMA) reporting guidelines [7].

### 2.2. Search Strategy

A systematic literature search was conducted in August 2024 across the Medline (via PubMed), EMBASE, and Web of Science databases. The search was finalised on 16 August 2024 and covered all records published up to that date. The search was restricted to studies published in English. The complete search strategy is provided in the Supplementary Material S1. Further database searches were performed on 6 June 2025, and no further studies were identified. Grey literature sources were also reviewed to identify additional relevant studies. Additionally, manual searches of the reference lists of all included studies were performed to identify any studies missed during the electronic search.

### 2.3. Inclusion Criteria

English-language studies were evaluated for inclusion if they examined the application of radiomics in predicting the malignant transformation of sinonasal inverted papilloma. Case reports, case series, and conference abstracts were excluded from the analysis.

- Study design:
  - Cohort studies.
  - Original research (>20 patients).
- Participants:
  - Patients with sinonasal inverted papilloma.
- Intervention:
  - Radiomic signature development.
- Outcomes:
  - Ability to predict malignant transformation.

### 2.4. Study Selection, Data Extraction, and Critical Appraisal

A reference database was established in EndNote X9™ reference management software. Two investigators (CW and HT) independently screened search results for eligibility.

**Figure 1:**
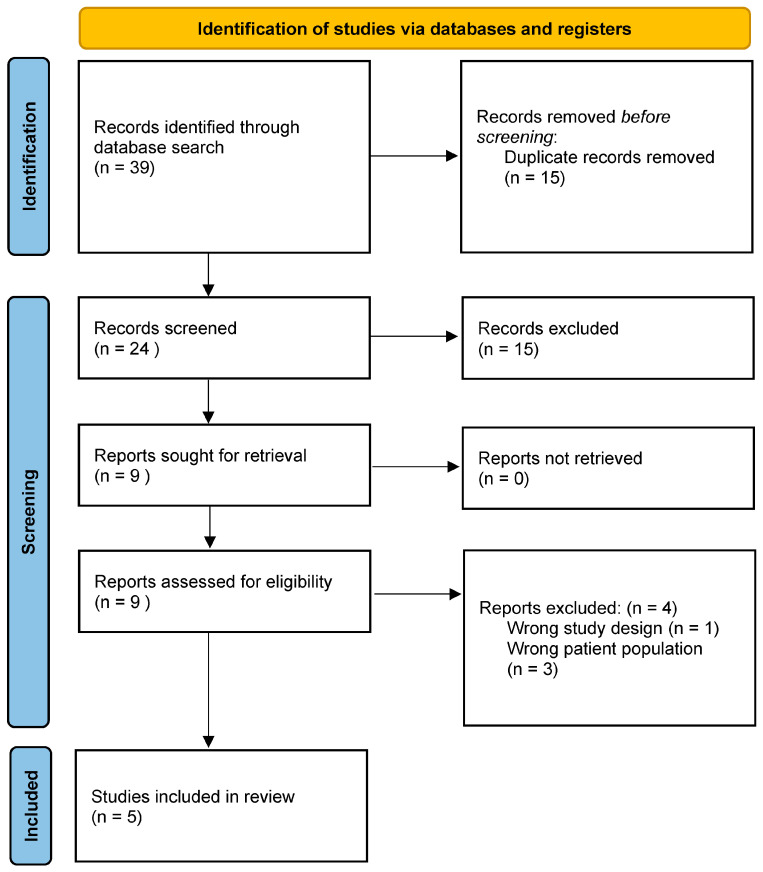
PRISMA flowchart.

Duplicate records were first eliminated, followed by title screening for relevance. Abstracts of potentially relevant studies were evaluated against the predefined inclusion/exclusion criteria. Excluded studies were categorised in the database according to exclusion rationale. Full-text reviews of eligible abstracts were then conducted, applying the same criteria. The PRISMA flow diagram (Figure 1) provides a comprehensive summary of the study selection process.

Data extraction and management were streamlined using Covidence, the Cochrane Collaboration’s screening tool [8]. Two reviewers (CW and HT) independently collected data under standardised headings: study details, design, population, interventions, comparators, and outcomes. Discrepancies in study selection or data extraction were resolved through open discussion to reach consensus.

The methodological quality and risk of bias of the included studies were critically evaluated. Two reviewers (CW and HT) independently assessed each study using the QUADAS-2 (Quality Assessment of Diagnostic Accuracy Studies) tool and the Radiomics Quality Score (RQS) [9,10]. A third reviewer (AD) was asked to arbitrate in cases of discrepancies.

### 2.5. Statistical Analysis

Due to limitations in data quantity, a meta-analysis of included studies was not performed. Data has been presented qualitatively throughout the results.

### 2.6. Systematic Review Registration

Our systematic review was registered on PROSPERO in April 2025 (ID: CRD420251009391).

## 3. Results

### 3.1. Search Results

The systematic search identified 39 studies. After removing 15 duplicates, 24 abstracts underwent eligibility screening. Nine full-text articles were assessed, of which five fulfilled the inclusion criteria and were included in the final analysis.

### 3.2. Methodological Characteristics and Quality of Studies

All five analysed studies were retrospective cohort studies [6,11,12,13,14]. Methodological features of the included investigations are summarised in Table 1. Data quality, evaluated via RQS and QUADAS-2 assessments, were generally satisfactory. All studies were rated as low risk for bias according to QUADAS-2 criteria and achieved an RQS score of >27%. Detailed descriptions of these assessment tools and breakdown of results are provided in Supplementary Materials S2–S5.

### 3.3. Participant Characteristics

This analysis included 837 participants. Four studies included both training and test sets, while one study utilised a training set paired and two test sets. The training cohorts comprised 550 patients and test cohorts 287 patients. Key participant characteristics of the study populations are detailed in Table 2.

### 3.4. Acquisition Parameters

Magnetic resonance imaging (MRI) was the imaging modality employed by all of the studies included in this analysis. Full acquisition parameters are illustrated in Table 3.

### 3.5. Development of Signatures

Whilst precise radiomic feature-extraction methods varied across the included studies, there were similarities to be found between them. They all emphasise the use of advanced software tools, such as 3D Slicer with PyRadiomics (v4.13), ITK-SNAP (v3.8.0), Python-based environments and CNNs (v3.8) for delineating regions of interest (ROI) and extracting meaningful features. These ROIs were manually identified by experienced radiologists and otolaryngologists in the studies. Intra-observer and interobserver variability were quantified in three studies using the intraclass correlation coefficient (ICC), with reported values ranging from 0.75 to 0.9, reflecting acceptable to excellent reliability of radiomic feature extraction.

In the study by Liu et al. [11] the implementation of 3D CNNs was used for feature extraction and their diagnostic signature due to their ability to automatically extract rich, high-level features from the images without manual intervention. Three studies [12,13,14] combined both radiomic features with clinical features in the development of their signatures. In comparison, Ramkumar et al. [6] utilised texture analysis with machine learning to achieve their diagnostic signature. Table 4 presents a breakdown of individual signatures and overall findings as well as the area under the curve (AUC) and the accuracy of signatures for predicting primary outcomes. Various feature selection techniques are employed across the studies to refine and reduce the feature sets, ensuring that only the most relevant and reproducible features are used for machine learning models. The specific software, feature selection techniques, and image processing utilised in each study are demonstrated in Table 5.

### 3.6. Performance of Signatures

Model performance, assessed using receiver operating characteristic (ROC) curve analysis and reported as the area under the curve (AUC), varied across the included studies. Another means of estimating the performance of the models was assessing the accuracy of the signature. All included studies had satisfactory performance in identifying the morphological change/malignant transformation of IP. In regard to Liu et al. [11], the focus varies between handcrafted features, which are predefined and require domain expertise, and deep learning features, which are automatically extracted CNNs. The CNN-based approaches, particularly in MRI data for predicting malignant transformations, demonstrate superior performance by eliminating the need for manual feature selection. All five studies reported a substantial improvement in performance upon incorporation of combined radiomics features and use of CNNs in the developed signature.

## 4. Discussion

This review synthesises current evidence on the development and performance of quantitative imaging models designed to predict malignant transformation in sinonasal inverted papillomas. The included investigations demonstrated generally satisfactory predictive accuracy in the validation cohorts, with area under the curve values (AUCs) ranging from 0.8–0.989. A methodological appraisal using the QUADAS-2 and Radiomics Quality Score (RQS) tools indicated satisfactory overall quality and low risk of bias, with most studies receiving high RQS ratings. These findings suggest that the methods and reporting practices in the included research compare favourably to similar radiomics studies, thereby enhancing confidence in the reliability of the reported outcomes [15,16,17].

Although radiomics-based signatures for predicting malignant transformation in sinonasal inverted papillomas remain understudied, an increasing number of publications have highlighted their utility in differential diagnosis, predicting attachment, recurrence-free survival, and risk factors [18,19,20,21]. Although no similar reviews assessing radiomics performance in predicting malignant transformation of benign aetiologies could be found, reviews have been conducted that look at radiomics performance in differentiating between benign and malignant pathologies. Harding-Theobald et al. demonstrated good discriminatory performance when radiomic features were used to differentiate hepatocellular carcinoma from other solid lesions (c-statistics 0.66–0.95) in their review of 54 studies [22].

The integration of radiomics into real-world clinical practice for sinonasal inverted papilloma has the potential to significantly alter surgical and perioperative decision-making by providing accurate, non-invasive preoperative prediction of malignant transformation. When malignancy is predicted preoperatively, this information may prompt surgeons to pursue more extensive surgical resection with wider margins, adjust intraoperative frozen section strategies to critical structures such as the orbit, or proceed with elective neck dissection in cases where the risk of occult nodal metastasis is elevated, as is standard for sinonasal squamous cell carcinoma [5,12,13]. Radiomics-based risk stratification can also improve patient counselling and multidisciplinary planning, allowing for tailored surgical approaches and more appropriate allocation of resources for intraoperative pathology [5,12]. Furthermore, the use of radiomics models has been shown to outperform experienced radiologists in differentiating IP from IP-SCC, supporting its role as a decision-support tool that can reduce interobserver variability and enhance diagnostic confidence [5,12,13]. As such, the adoption of radiomics in this context is likely to drive a shift toward more personalised, evidence-based management of sinonasal IP, with direct implications for surgical extent, intraoperative assessment, and the decision to perform elective neck dissection when malignancy is predicted.

Despite encouraging preliminary findings, the substantial heterogeneity among the included studies, coupled with a paucity of prospective validation, remains a primary limitation. Standardisation through open-source imaging datasets, segmentation protocols, and analytic code is essential to enable controlled external validation across multiple institutions [9]. Ultimately, prospective validation—preferably conducted in a multicentre framework—is paramount for clinical implementation, ensuring that inter-institutional variability is addressed and strengthening the predictive accuracy of the proposed quantitative imaging models [23,24].

A central challenge in radiomics research arises from deficiencies in repeatability and reproducibility and the heterogeneity of feature extraction methods [25]. These issues are further complicated by variations in image acquisition protocols and segmentation procedures across different institutions. “Repeatability” refers to repeated measurements of the same or closely related parameters under identical or nearly identical conditions—encompassing the same operators, measurement systems, and physical locations within a short timeframe. In contrast, “reproducibility” involves obtaining repeated measurements under varying conditions, including different locations, operators, or scanners [26,27]. Addressing these limitations requires standardised imaging protocols, rigorous quality assurance practices, and uniform acquisition parameters to ensure the reliable integration of radiomics into clinical settings.

In evaluations of radiomic feature repeatability, Balagurunathan et al. [28] observed that 30.14% (66 out of 219) of the features exhibited strong concordance correlation coefficients and acceptable dynamic ranges in lung CT image features for patients with non-small-cell lung cancer. Similarly, Berenguer et al. [29] reported a test–retest repeatability ratio of 91% (161 out of 177 radiomic features) in CT radiomic features.

In a recent review, Pfaehler et al. [30] identified the intraclass correlation coefficient (ICC) as the most used metric for quantifying reproducibility, documenting its application in 19 human-based and 4 phantom-based studies. Their findings emphasised the fact that variations in image acquisition, reconstruction methods, preprocessing, and discretisation substantially impact radiomic features [30]. Park et al. further demonstrated the enhanced reproducibility of CT features using a convolutional neural network (CNN)-based super-resolution algorithm [31].

Prior research has aimed to identify radiomic feature subtypes that consistently exhibit high reproducibility, thereby informing subsequent radiomics investigations. For example, Berenguer et al. reported that 71 of 177 evaluated features—derived from five different CT scanners—met reproducibility criteria, although only 10 remained clinically relevant [29]. Park et al. [31] proposed strategies for maximizing reproducibility and repeatability, emphasizing the importance of improving intra-individual repeatability and reproducibility, multi-scanner and multi-institution reproducibility, and multi-reader reproducibility and for optimizing both imaging reconstruction and processing protocols. Additionally, they suggested that intra-individual test–retest studies using phantoms or patient data across CT, MRI, and PET modalities can further enhance repeatability [31]. Nonetheless, reproducibility challenges persist in MRI-based radiomics due to differences in signal intensity and non-standardised pixel values [32].

A primary limitation of this review is the small number of studies included. Moreover, all of these studies were retrospective, introducing a potential selection bias. While each investigation used MRI-based radiomics to develop its model, variations in study design and analytic methods may have influenced the results and their subsequent interpretation. Heterogeneity in primary outcomes also impeded any meta-analysis. A further limitation is the inconsistent reporting across included studies regarding whether the diagnosis of inverted papilloma and exclusion of malignancy were confirmed preoperatively, limiting our ability to fully assess the true predictive value of radiomics in the pre-surgical setting. Additionally, the geographic concentration of three of the five studies in a single region may limit the generalizability of findings across diverse populations or settings. Nonetheless, given that radiomics remains an emerging field, constraints related to study numbers and geographic concentration are often inevitable. Despite these challenges, the consistent performance of radiomic signatures, coupled with the low risk of bias indicated by the QUADAS-2 tool, points to considerable predictive potential.

## 5. Conclusions

Radiomics-driven nomograms and deep learning approaches show promising potential in predicting the malignant transformation of sinonasal inverted papillomas, with included studies demonstrating high predictive accuracy, and could be used in the future as a non-invasive adjunct for clinical decision making. Future research should emphasise standardising methodologies and MRI protocols and the use of open-source datasets for the validation of radiomics models for predicting malignant transformation in sinonasal inverted papillomas in larger, multicentre cohorts.

## Figures and Tables

**Table 1:** Methodological characteristics of the included studies.

Study	Country	Journal	Study Design	Primary Outcome
Liu 2022 [11]	USA	International Forum of Allergy & Rhinology	Retrospective, Multicentre	Classification of IP malignant transformation using 3D CNNs
Xia 2024 [12]	China	Clinical Radiology	Retrospective, Single centre	Prediction of malignant transformation in IP based on MR radiomics and clinical risk factors.
Gu 2022 [13]	China	Frontiers in Oncology	Retrospective, Single centre	Differentiation of SNIP and MST using MR radiomics models
Yan 2022 [14]	China	Frontiers in Oncology	Retrospective, Single centre	Preoperative prediction of the malignant transformation of IP using MR radiomics
Ramkumar 2017 [6]	USA	American Journal of Neuroradiology	Retrospective, Single centre	MRI-Based texture analysis to differentiate IP from IP-SCC

IP: inverted papilloma, CNN: convolutional neural networks, MR: magnetic resonance, SNIP: sinonasal inverted papilloma, MST: malignant sinonasal tumours, SCC: squamous cell carcinoma.

**Table 2:** Participant characteristics.

Study	No. Patients		Age	Dataset Splitting	M:F
Liu 2022 [11]	64 IP	26 IP-SCC	Mean age: IP: 59.7, IP-SCC: 62.9	72 (training), 18 (test)	IP: 45:19 IP-SCC: 18:8
Xia 2024 [12]	143 IP	75 IP-SCC	Mean age: Training: 56.10, Test: 59.18	153 (training), 65 (test)	Training: 110:43 Test: 48:17
Gu 2022 [13]	106 SNIP	141 MST	Mean age: IP: 58.1, SCC: 54.2	135 (training), 58 (test 1), 54 (test 2)	Training: 93:42 Test 1: 40:18 Test 2: 35:19
Yan 2022 [14]	144 IP	92 IP-SCC	Not specified	157 (training), 79 (test)	IP: 97:47 IP-SCC: 72:20
Ramkumar 2017 [6]	22 IP	24 IP-SCC	Mean age: IP: 58.1, SCC: 54.2	33 (training), 13 (test)	IP: 17:5 IP-SCC: 19:5

IP: inverted papilloma, SCC: squamous cell carcinoma, SNIP: sinonasal inverted papilloma, MST: malignant sinonasal tumour.

**Table 3:** MRI scanning parameters.

Study	Phase	Model	Field Strength	FOV (cm^2^)	TE/TR (ms)	ST (mm)	SI (mm)	Matrix	Contrast
Liu 2022 [11]	T1WI CE-T1WI T2WI	-	1.5–3.0 T	-	-	-	-	-	Gadolinium enhancement
Xia 2024 [12]	T2WI DWI CE-T1WI	Magnetom Verio	3 T	18–22 × 18–22	384–4700/9–99	6 mm 3 mm 5 mm	0.6 mm 1.2 mm 0.6 mm	640 × 592 160 × 160 640 × 592	0.1 mmol/kg Magnevist at 2 mL/s.
Gu 2022 [13]	T1WI FSE T2WI CE-T1WI	Magnetom Essenza Magnetom Skyra GE Signa HDxt	1.5–3.0 T	22–24 × 22–24	400–4260/10–93	4–5 mm	1 mm	256 × 204 288 × 224 320 × 224	0.1 mL/kg Gd⁃DTPA and rate of 2.5 mL/s
Yan 2022 [14]	T1WI FSE T2WI FSE CE-T1WI T1WI TSE T2WI TSE	Ingenia GE Signa-HDxt	3 T	21–22 × 19–22	400–4720/6–90	4–5 mm	0.3–0.5 mm	264–512 × 201–256	0.1 mL/kg Gd-DTPA
Ramkumar 2017 [6]	T1WI FSE T2WI FSE CE-T1WI FSE	-	1.5–3.0 T	≤20	-	≤5 mm	-	256 × 192	Gadolinium enhancement

T1WI: T1 weighted image, CE-T1WI: contrast-enhanced T1 weighted image, T2WI: T2 weighted image, FSE: fast spin echo, TSE: turbo spin echo.

**Table 4:** Software and Performance.

Study	Segmentation Software	Radiomics Software	Performance of Signature (Training)					Performance of Signature (Test)					
			AUC	Accuracy	Sens	Spec	95% CI	AUC		Accuracy	Sens	Spec	95% CI
Liu 2022 [11]	-	Training performed in PyTorch 1.8	-	-	-	-	-	0.80		77.9%	0.667	0.815	0.68–0.9
Xia 2024 [12]	ITK-SNAP (v3.8.0) 3D Slicer (v4.11)	PyRadiomics v3.0.1	0.987	-	-	-	0.975–1.00	0.989		-	-	-	0.973–1.00
Gu 2022 [13]	ITK-SNAP (v3.6.0)	PyRadiomics v3.0.1	0.901	81.5%	0.831	0.879	0.837–0.946	Test set 1	0.878	0.788	0.788	0.800	0.765–0.949
								Test set 2	0.914	0.935	0.833	0.696	0.806–0.973
Yan 2022 [14]	ITK-SNAP (v3.6.0)	PyRadiomics (v3.0.1)	0.954	87.3%	0.857	0.883	0.926–0.982	0.940		0.873	0.793	0.920	0.888–0.992
Ramkumar 2017 [6]	-	R statistical and computing software, Python 2.7+custom code	-	90.9%	0.941	0.875	-	-		84.6%	0.857	0.833	-

AUC: area under curve, CI: confidence interval.

**Table 5:** Feature processing and nomogram construction.

Study	Pre-Processing	Feature Selection Process	Selected Features	Nomogram/CNN Models
Liu 2022 [11]	Image volumes were resampled to a size of 128 × 128 × 64 voxels and normalised	Manual segmentation of 16 × 16-pixel regions of interest Feature extraction with CNNs	446 images of distinct MRI sequences	3D CNNs trained using backpropagation and Adam-optimized stochastic gradient descent: All-Net, Small-All-Net, and Elastic-All-Net
Xia 2024 [12]	Resampling and signal intensity normalisation Z-score standardisation	LASSO Minimum redundancy maximum relevance Pearson’s or Spearman’s correlation Statistical testing	CE wavelet-LLH GLSZM zone entropy, T2 wavelet-HLH glcm Idmn, ADC original first order skewness, CE wavelet-LLL first order interquartile range, T2 wavelet-LHH first order kurtosis, CE original shape maximum 2D diameter row, ADC wavelet-LHL GLSZM grey level non-uniformity, CE wavelet-LHL first order maximum, CE wavelet-LLH GLDM large dependence high grey level emphasis, CE original shape flatness, T2 original first order 10 percentile, ADC original first order 10 percentile	Clinical and radiomics model: (1.188 × epistaxis) + (2.503 × T2 equal signal) + (0.675 × extranasal invasion) + (0.978 × loss of CCP) + (2.899 × rad-score)—1.458
Gu 2022 [13]	Z-score standardisation Images resampled to a consistent voxel spacing of 1 × 1 × 1 mm	Spearman correlation coefficients 10-fold cross-validation Recursive feature elimination	1130 radiomics features were extracted: wavelet-LLH_firstorder_10Percentile, wavelet-LHL_firstorder_Mean, wavelet-LHL_glszm_SmallAreaLowGrayLevelEmphasis, wavelet-LHH_glszm_SmallAreaEmphasis, wavelet-HHH_glcm_Idm, wavelet-LLL_glcm_MCC, log-sigma-1-0-mm-3D_firstorder_10Percentile, log-sigma-1-0-mm-3D_firstorder_90Percentile, log-sigma-2-0-mm-3D_glcm_ClusterShade, log-sigma-2-0-mm-3D_glszm_SmallAreaLowGrayLevelEmphasis, log-sigma-3-0-mm-3D_firstorder_Median, log-sigma-3-0-mm-3D_firstorder_RootMeanSquared, log-sigma-3-0-mm-3D_glcm_ClusterShade, log-sigma-3-0-mm-3D_glcm_Correlation, log-sigma-3-0-mm-3D_glcm_MaximumProbability, log-sigma-3-0-mm-3D_glszm_LargeAreaLowGrayLevelEmphasis, log-sigma-3-0-mm-3D_glszm_SmallAreaEmphasis, log-sigma-3-0-mm-3D_ngtdm_Complexity	Combination of radiomics features and clinic-radiological features
Yan 2022 [14]	B-Spline interpolation Z-score standardisation	Multivariable logistic regression Support vector machine Minimum redundancy Maximum relevance	A total of 3948 radiomic features: squareroot_glszm_SizeZoneNonUniformity, wavelet. HLL_glrlm_RunEntropy, square_ngtdm_Coarseness, gradient_glszm_SizeZoneNonUniformityNormalized, original_shape_SurfaceVolumeRatio, logarithm_glcm_ClusterProminenc, logarithm_firstorder_Minimum, logarithm_glszm_SizeZoneNonUniformity, logarithm_glcm_Contrast, squareroot_ngtdm_Complexity, logarithm_glszm_SizeZoneNonUniformity, logarithm_glcm_DifferenceVariance, square_firstorder_Minimum, logarithm_glszm_LargeAreaLowGrayLevelEmphasis, logarithm_glszm_GrayLevelNonUniformityNormalized	MR radiomic features and morphological features
Ramkumar 2017 [6]	Resampling and/or zero-padding and normalisation	Manual ROI extraction Sequential forward-feature selection 16 × 16 square ROIs	231 texture features were calculated for each case (77 texture features per MR imaging contrast × 3 contrasts) 13 GLCM features 12 LBP features 10 DOST features 18 LoGHist features 8 GFB features	Texture analysis integrated into machine-learning models

CNN: convoluted neural network, MRI: magnetic resonance imaging, LASSO: least absolute shrinkage and selection operator, CE: contrast enhanced, GLSZM: gray level size zone, IDMN: inverse difference moment normalised, ADC: apparent diffusion coefficient, GLDM: gray level dependence matrix, CCP: convoluted cerebriform pattern, ROI: region of interest, MR: magnetic resonance, GLCM: gray level co-occurrence matrix, LBP: local binary pattern, DOST: Discrete Orthonormal Stockwell Transform, LoGHist: Laplacian of Gaussian Histogram, GFB: Gabor Filter Banks.

## Data Availability

The original data presented in the study is openly available via the Medline, Embase, and Web of Science databases.

## Supplementary Materials

The following supporting information can be downloaded at https://www.mdpi.com/article/10.3390/cancers17132175/s1, Supplementary Material S1: Search strategy; Supplementary Material S2: Description of the radiomics quality score (RQS) tool; Supplementary Material S3: Description of the revised Quality Assessment of Diagnostic Accuracy Studies (QUADAS-2) tool; Supplementary Material S4: Methodological quality assessment of each study by the RQS tool; Supplementary Material S5: Risk of bias and application concerns assessment of each study by the QUADAS-2 tool.

## References

[1] Viitasalo S., Ilmarinen T., Aaltonen L., Hagström J., Hytönen M., Hammarén-Malmi S., Pietarinen P., Järvenpää P., Kinnari T., Geneid A. (2023). Sinonasal inverted papilloma—Malignant transformation and non-sinonasal malignancies. Laryngoscope.

[2] Mendenhall W.M., Hinerman R.W., Malyapa R.S., Werning J.W., Amdur R.J., Villaret D.B., Mendenhall N.P. (2007). Inverted papilloma of the nasal cavity and paranasal sinuses. Am. J. Clin. Oncol..

[3] Melroy C.T., Senior B.A. (2006). Benign sinonasal neoplasms: A focus on inverting papilloma. Otolaryngol. Clin. North Am..

[4] Mirza S., Bradley P.J., Acharya A., Stacey M., Jones N.S. (2007). Sinonasal inverted papillomas: Recurrence, and synchronous and metachronous malignancy. J. Laryngol. Otol..

[5] Yan C.H., Tong C.C.L., Penta M., Patel V.S., Palmer J.N., Adappa N.D., Nayak J.V., Hwang P.H., Patel Z.M. (2019). Imaging predictors for malignant transformation of inverted papilloma. Laryngoscope.

[6] Ramkumar S., Ranjbar S., Ning S., Lal D., Zwart C., Wood C., Weindling S., Wu T., Mitchell J., Li J. (2017). MRI-Based Texture Analysis to Differentiate Sinonasal Squamous Cell Carcinoma from Inverted Papilloma. AJNR Am. J. Neuroradiol..

[7] Page M.J., McKenzie J.E., Bossuyt P.M., Boutron I., Hoffmann T.C., Mulrow C.D., Shamseer L., Tetzlaff J.M., Akl E.A., Brennan S.E. (2021). The PRISMA 2020 statement: An updated guideline for reporting systematic reviews. BMJ.

[8] Veritas Health Innovation (2014). Covidence Systematic Review Software.

[9] Whiting P.F., Rutjes A.W.S., Westwood M.E., Mallett S., Deeks J.J., Reitsma J.B., Leeflang M.M.G., Sterne J.A.C., Bossuyt P.M.M., QUADAS-2 Group (2011). QUADAS-2: A revised tool for the quality assessment of diagnostic accuracy studies. Ann. Intern. Med..

[10] Lambin P., Leijenaar R.T.H., Deist T.M., Peerlings J., de Jong E.E.C., van Timmeren J., Sanduleanu S., Larue R.T.H.M., Even A.J.G., Jochems A. (2017). Radiomics: The bridge between medical imaging and personalized medicine. Nat. Rev. Clin. Oncol..

[11] Liu G.S., Yang A., Kim D., Hojel A., Voevodsky D., Wang J., Tong C.C., Ungerer H., Palmer J.N., Kohanski M.A. (2022). Deep learning classification of inverted papilloma malignant transformation using 3D convolutional neural networks and magnetic resonance imaging. Int. Forum Allergy Rhinol..

[12] Xia Z., Lin N., Chen W., Qi M., Sha Y. (2024). Multiparametric MRI-based radiomics nomogram for predicting malignant transformation of sinonasal inverted papilloma. Clin. Radiol..

[13] Gu J., Yu Q., Li Q., Peng J., Lv F., Gong B., Zhang X. (2022). MRI radiomics-based machine learning model integrated with clinic-radiological features for preoperative differentiation of sinonasal inverted papilloma and malignant sinonasal tumors. Front. Oncol..

[14] Yan Y., Liu Y., Tao J., Li Z., Qu X., Guo J., Xian J. (2022). Preoperative Prediction of Malignant Transformation of Sinonasal Inverted Papilloma Using MR Radiomics. Front. Oncol..

[15] Barry N., Kendrick J., Molin K., Li S., Rowshanfarzad P., Hassan G.M., Dowling J., Parizel P.M., Hofman M.S., Ebert M.A. (2025). Evaluating the impact of the Radiomics Quality Score: A systematic review and meta-analysis. Eur. Radiol..

[16] Huang M.-L., Ren J., Jin Z.-Y., Liu X.-Y., He Y.-L., Li Y., Xue H.-D. (2023). A systematic review and meta-analysis of CT and MRI radiomics in ovarian cancer: Methodological issues and clinical utility. Insights Into Imaging.

[17] Stanzione A., Gambardella M., Cuocolo R., Ponsiglione A., Romeo V., Imbriaco M. (2020). Prostate MRI radiomics: A systematic review and radiomic quality score assessment. Eur. J. Radiol..

[18] Du L., Yuan Q., Han Q. (2023). A new biomarker combining multimodal MRI radiomics and clinical indicators for differentiating inverted papilloma from nasal polyp invaded the olfactory nerve possibly. Front. Neurol..

[19] He S., Zhao Y., Shi L., Yang X., Wang X., Luo Y., Wang M., Zhang X., Li X., Yu D. (2024). Utilizing radiomics for differential diagnosis of inverted papilloma and chronic rhinosinusitis with polyps based on unenhanced CT scans. Sci. Rep..

[20] McKee S.P., Liang X., Yao W.C., Anderson B., Ahmad J.G., Allen D.Z., Hasan S., Chua A.J., Mokashi C., Islam S. (2025). Predicting sinonasal inverted papilloma attachment using machine learning: Current lessons and future directions. Am. J. Otolaryngol..

[21] Miao S., Cheng Y., Li Y., Chen X., Chen F., Zha D., Xue T. (2024). Prediction of recurrence-free survival and risk factors of sinonasal inverted papilloma after surgery by machine learning models. Eur. J. Med. Res..

[22] Harding-Theobald E., Louissaint J., Maraj B., Cuaresma E., Townsend W., Mendiratta-Lala M., Singal A.G., Su G.L., Lok A.S., Parikh N.D. (2021). Systematic review: Radiomics for the diagnosis and prognosis of hepatocellular carcinoma. Aliment. Pharmacol. Ther..

[23] Liu F., Ning Z., Liu Y., Liu D., Tian J., Luo H., An W., Huang Y., Zou J., Liu C. (2018). Development and validation of a radiomics signature for clinically significant portal hypertension in cirrhosis (CHESS1701): A prospective multicenter study. eBioMedicine.

[24] Ibrahim A., Primakov S., Beuque M., Woodruff H., Halilaj I., Wu G., Refaee T., Granzier R., Widaatalla Y., Hustinx R. (2021). Radiomics for precision medicine: Current challenges, future prospects, and the proposal of a new framework. Methods.

[25] Mao W., Zhou J., Zhang H., Qiu L., Tan H., Hu Y., Shi H. (2019). Relationship between KRAS mutations and dual time point ^18^F-FDG PET/CT imaging in colorectal liver metastases. Abdom. Radiol..

[26] Raunig D.L., McShane L.M., Pennello G., Gatsonis C., Carson P.L., Voyvodic J.T., Wahl R.L., Kurland B.F., Schwarz A.J., Gönen M. (2015). Quantitative imaging biomarkers: A review of statistical methods for technical performance assessment. Stat. Methods Med. Res..

[27] Kessler L.G., Barnhart H.X., Buckler A.J., Choudhury K.R., Kondratovich M.V., Toledano A., Guimaraes A.R., Filice R., Zhang Z., Sullivan D.C. (2015). The emerging science of quantitative imaging biomarkers terminology and definitions for scientific studies and regulatory submissions. Stat. Methods Med. Res..

[28] Balagurunathan Y., Kumar V., Gu Y., Kim J., Wang H., Liu Y., Goldgof D.B., Hall L.O., Korn R., Zhao B. (2014). Test-retest reproducibility analysis of lung CT image features. J. Digit. Imaging.

[29] Berenguer R., del Rosario Pastor-Juan M., Canales-Vazquez J., Castro-García M., Villas M.V., Masilla Legorburo F., Sabater S. (2018). Radiomics of CT Features May Be Nonreproducible and Redundant: Influence of CT Acquisition Parameters. Radiology.

[30] Pfaehler E., Zhovannik I., Wei L., Boellaard R., Dekker A., Monshouwer R., El Naqa I., Bussink J., Gillies R., Wee L. (2021). A systematic review and quality of reporting checklist for repeatability and reproducibility of radiomic features. Phys. Imaging Radiat. Oncol..

[31] Park J.E., Park S.Y., Kim H.J., Kim H.S. (2019). Reproducibility and Generalizability in Radiomics Modeling: Possible Strategies in Radiologic and Statistical Perspectives. Korean J. Radiol..

[32] Kumar V., Gu Y., Basu S., Berglund A., Eschrich S.A., Schabath M.B., Forster K., Aerts H.J.W.L., Dekker A., Fenstermacher D. (2012). Radiomics: The process and the challenges. Magn. Reson. Imaging.

