# Supplementary material for: Role of Radiomics to Predict Malignant Transformation of Sinonasal Inverted Papilloma: A Systematic Review
Source: Cancers (Basel). 2025 Jun 27;17(13):2175. doi: 10.3390/cancers17132175 (PMC12249154; doi:10.3390/cancers17132175)
Supplement: Supplementary file 1 [file cancers-17-02175-s001.zip › cancers-3676108-supplementary.pdf]

Supplementary Material S1: Search strategy.

("radiomic*" OR "radiogenomic*" OR "radiomic features" OR "radiogenomic analysis" OR "texture analysis" OR "quantitative imaging")

AND

("sinonasal inverted papilloma*" OR "inverted papilloma*" OR "Schneiderian papilloma*" OR "SNIP" OR "sinonasal tumour*" OR "sinonasal neoplasm*")

AND

("malignant transformation" OR "malignant progression" OR "malignant change" OR "carcinoma" OR "squamous cell carcinoma" OR "transformation" OR "neoplastic progression")

Supplementary Material S2: Description of the radiomics quality score (RQS) tool.

	Criteria	Points
1	Image protocol quality - well-documented image protocols (for example, contrast, slice thickness, energy, etc.) and/or usage of public image protocols allow reproducibility/replicability	+1 (if protocols are well documented) +1 (if public protocol is used)
2	Multiple segmentations - possible actions are: segmentation by different physicians/algorithms/software, perturbing segmentations by (random) noise, segmentation at different breathing cycles. Analyse feature robustness to segmentation variabilities	+1
3	Phantom study on all scanners - detect interscanner differences and vendor-dependent features. Analyse feature robustness to these sources of variability	+1
4	Imaging at multiple time points - collect images of individuals at additional time points. Analyse feature robustness to temporal variabilities (for example, organ movement, organ expansion/ shrinkage)	+1
5	Feature reduction or adjustment for multiple testing - decreases the risk of overfitting. Overfitting is inevitable if the number of features exceeds the number of samples. Consider feature robustness when selecting features	-3 (if neither measure is implemented)<br>+3 (if either measure is implemented)
6	Multivariable analysis with non radiomics features (for example, EGFR mutation) - is expected to provide a more holistic model. Permits correlating/inferencing between radiomics and non radiomics features	+1
7	Detect and discuss biological correlates - demonstration of phenotypic differences (possibly associated with underlying gene–protein expression patterns) deepens understanding of radiomics and biology	+1
8	Cut-off analyses - determine risk groups by either the median, a previously published cut-off or report a continuous risk variable. Reduces the risk of reporting overly optimistic results	+1
9	Discrimination statistics - report discrimination statistics (for example, C-statistic, ROC curve, AUC) and their statistical significance (for example, p-values, confidence intervals). One can also apply resampling method (for example, bootstrapping, cross-validation)	+1 (if a discrimination statistic and its statistical significance are reported)<br>+1 (if a resampling method technique is also applied)
10	Calibration statistics - report calibration statistics (for example, Calibration-in-thelarge/slope, calibration plots) and their statistical significance (for example, P-values, confidence intervals). One can also apply resampling method (for example, bootstrapping, cross-validation)	+1 (if a calibration statistic and its statistical significance are reported)<br>+1 (if a resampling method technique is also applied)
11	Prospective study registered in a trial database - provides the highest level of evidence supporting the clinical validity and usefulness of the radiomics biomarker	+7 (for prospective validation of a radiomics signature in an appropriate trial)
12	Validation - the validation is performed without retraining and without adaptation of the cut-off value, provides crucial information with regard to credible clinical performance	-5 (if validation is missing)<br>+2 (if validation is based on a dataset from the same institute) +3 (if validation is based on a dataset from another institute)

		+4 (if validation is based on two datasets from two distinct institutes)<br>+4 (if the study validates a previously published signature)<br>+5 (if validation is based on three or more datasets from distinct institutes)<br>*Datasets should be of comparable size and should have at least 10 events per model feature
13	Comparison to ‘gold standard’ - assess the extent to which the model agrees with/is superior to the current ‘gold standard’ method (for example, TNM-staging for survival prediction). This comparison shows the added value of radiomics	+2
14	Potential clinical utility - report on the current and potential application of the model in a clinical setting (for example, decision curve analysis).	+2
15	Cost-effectiveness analysis - report on the cost-effectiveness of the clinical application (for example, QALYs generated)	+1
16	Open science and data - make code and data publicly available. Open science facilitates knowledge transfer and reproducibility of the study	+1 (if scans are open source)<br>+1 (if region of interest segmentations are open source)<br>+1 (if code is open source)<br>+1 (if radiomics features are calculated on a set of representative ROIs and the calculated features andrepresentative ROIs are open source)
Total points (36=100%)		

Source: https://www.radiomics.world/rqs

Supplementary Material S3: Description of the revised Quality Assessment of Diagnostic Accuracy Studies (QUADAS-2) tool.

Domain	Patient selection	Index test	Reference standard	Flow and timing
Signalling questions (yes, no, or unclear)	Was a consecutive or random sample of patients enrolled? Was a case-control design avoided? Did the study avoid inappropriate exclusions?	Were the index test results interpreted without knowledge of the results of the reference standard? If a threshold was used, was it prespecified?	Is the reference standard likely to correctly classify the target condition? Were the reference standard results interpreted without knowledge of the results of the index test?	Was there an appropriate interval between index test and reference standard? Did all patients receive a reference standard? Did all patients receive the same reference standard? Were all patients included in the analysis?
Risk of bias (high, low, or unclear)	Could the selection of patients have introduced bias?	Could the conduct or interpretation of the index test have introduced bias?	Could the reference standard, its conduct, or its interpretation have introduced bias?	Could the patient flow have introduced bias?
Concerns about applicability (high, low, or unclear)	Are there concerns that the included patients do not match the review question?	Are there concerns that the index test, its conduct, or its interpretation differ from the review question?	Are there concerns that the target condition as defined by the reference standard does not match the review question?	-

Source: Whiting PF, Rutjes AW, Westwood ME, et al; QUADAS-2 Group. QUADAS-2: a revised tool for the quality assessment of diagnostic accuracy studies. Ann Intern Med. 2011 Oct 18;155(8):529-36. doi: 10.7326/00

Supplementary Material S4: Methodological quality assessment of each study by the RQS tool.

Study	Image protocol quality	Multiple segmentations	Phantom study	Imaging at multiple time points	Feature reduction	Multivariable analysis with non-radiomic features	Biological correlates	Cut-off analyses	Discrimination statistics	Calibration statistics	Prospective study	Validation	Comparison to 'gold standard'	Potential clinical utility	Cost-effectiveness analysis	Open science and data	Total points (/36)
Liu et al. 2022	2	1	0	0	3	0	1	0	2	1	0	2	0	0	0	0	11 (30.6%)
Xia et al. 2024	2	1	0	0	0	0	1	0	1	2	0	2	0	2	0	0	10 (27.8%)
Gu et al. 2022	2	1	0	0	3	0	1	0	2	1	0	2	0	0	0	0	11 (30.6%)
Yan et al. 2022	2	1	0	0	0	0	1	0	2	1	0	2	2	0	0	0	10 (27.8%)
Ramkumar et al. 2017	2	1	0	0	0	0	1	0	2	1	0	2	2	0	0	0	10 (27.8%)

Supplementary Material S5: Risk of bias and application concerns assessment of each study by the QUADAS-2 tool.

	Risk of bias				Applicability Concerns		
Study ID	Patient selection	Index test	Reference standard	Flow and timing	Patient selection	Index test	Reference standard
Liu et al. 2022	Low	Low	Low	Low	Low	Low	Low
Xia et al. 2024	Low	Low	Low	Low	Low	Low	Low
Gu et al. 2022	Low	Low	Low	Low	Low	Low	Low
Yan et al. 2022	Low	Low	Low	Unclear	Low	Low	Low
Ramkumar et al. 2017	Low	Low	Low	Unclear	Low	Low	Low
